# Behavioral and autonomic manifestations of suspected focal impaired awareness seizures supported by interictal electroencephalographic findings in three dogs: a case series

**DOI:** 10.3389/fvets.2026.1904889

**Published:** 2026-08-28

**Authors:** Adriana Czerwik, Agnieszka Olszewska, Daniela Farke, Martin Jürgen Schmidt

**Affiliations:** 1 Department of Veterinary Clinical Sciences, Small Animal Clinic, Neurosurgery, Neuroradiology and Clinical Neurology, Justus Liebig University, Giessen, Germany; 2 Fachtierarztzentrum Cologne Merheim, Cologne, Germany

**Keywords:** behavioral signs, canine, EEG, epilepsy, focal seizure

## Abstract

Focal epileptic seizures with sensory impairment can be difficult to recognize and are often mistaken for behavioral disorders. Electroencephalography (EEG) can provide important supportive evidence for their epileptic origin when interpreted in conjunction with clinical presentation. This article describes three cases of canine patients: a 2-year-old Border Terrier, a 1-year-old Poodle mix, and a 4-year-old mixed-breed dog. All three were presented with a history of recurrent episodes characterized by fear, restlessness, hyperactivity, hiding, staring into space or at the sky, lip licking, and hypersalivation. In two dogs, there were also signs of impaired consciousness. The frequency of the episodes varied, ranging from multiple episodes per day to one episode every 1–2 weeks. No pre-ictal or post-ictal signs were reported. Clinical and neurological examinations were unremarkable in all three cases. The diagnostic workup included EEG using a Natus Xltek system and magnetic resonance imaging (MRI) of the brain with a Siemens 1.5 T scanner. EEG monitoring lasting 1.5–4 h revealed epileptiform discharges predominantly in the parietal and temporal regions. This case series highlights the occurrence of behavioral and autonomic symptoms without typical motor manifestations as a presentation of presumed idiopathic epilepsy in dogs, supported by EEG findings in conjunction with the clinical presentation. All three dogs were treated with antiseizure medication, which resulted in a decrease in both the frequency and intensity of episodes, with complete seizure freedom achieved in two dogs.

## 1 Introduction

Epilepsy is one of the most common neurological diseases in dogs, with a reported prevalence of approximately 0.6–5.75%. Despite its clinical importance, many aspects of canine epilepsy remain less well understood than those of human epilepsy (1–3). In veterinary practice, diagnosing a dog with classic, generalized tonic–clonic seizures is generally straightforward. However, identifying and diagnosing cases involving hallucination-like behaviors, staring episodes, anxiety attacks, or autonomic signs remains a significant clinical challenge. Electroencephalography (EEG) is routinely used to evaluate individuals who present with seizures or atypical episodes, allowing for better classification and description of epileptic syndromes (4). However, the difficulty of performing EEGs in veterinary practice, combined with the inability to communicate directly with the patient, is likely the primary factor underlying the differences in epilepsy classification between humans and companion animals.

According to the International League Against Epilepsy (ILAE) and the Epilepsy Foundation, temporal lobe epilepsy is the most common form of focal epilepsy in humans (5, 6). Focal seizures are categorized based on the patient’s level of awareness. If awareness is preserved, the episode is termed a *focal aware seizure* (also known as an *aura*), while *focal impaired awareness seizures* involve altered consciousness. During an aura, patients may experience intense emotions such as fear, panic, or anxiety. These auras can evolve into focal impaired awareness seizures, during which individuals may appear to stare blankly, seem confused, or engage in repetitive behaviors such as lip-smacking or chewing (5, 6). However, in veterinary medicine, the term *aura* is not used (3). This is because such pre-ictal signs are difficult to identify with certainty in animals. Unlike humans, dogs cannot verbally communicate subjective sensations or early warning symptoms, making it nearly impossible to distinguish a true aura from other behaviors or environmental responses. For this reason, the concept of the aura is avoided in veterinary neurology. Instead, seizure activity is classified into three observable clinical stages: the prodrome, ictus, and postictal phases (3).

In veterinary medicine, epileptic motor activity can be either focal or generalized and is often accompanied by sensory disturbances that may mimic behavioral disorders (7). Differentiating between behavioral and epileptic episodes can be challenging and requires careful clinical assessment and appropriate diagnostic investigations (7). EEG can provide important supportive evidence for the epileptic origin of these atypical events when interpreted in conjunction with the clinical presentation.

This study aimed to present the neurological and electroencephalographic diagnostic workup of atypical behavioral incidents and/or autonomic signs in three dogs.

## 2 Case presentation

A 2-year-old Border Terrier, a 1-year-old Poodle mix, and a 4-year-old mixed-breed dog were presented to the Department of Veterinary Clinical Sciences at the Small Animal Clinic of Justus Liebig University, Giessen, Germany. The first case exhibited recurrent episodes of fear, restlessness, hyperactivity, rapid and repeated head turns to look behind or to the side, as if responding to an unseen stimulus, and sometimes snapped at the air without an apparent external stimulus. These episodes progressed to hiding, staring into space, and episodes of impaired consciousness resembling an absence seizure (Supplementary Video 1). The second case presented with frequent episodes of lip licking, staring into space, and fly-catching behavior, all accompanied by impaired consciousness (Supplementary Video 1).

The third case demonstrated recurrent episodes of restlessness, anxiety, and increased attachment to the owners, accompanied by motor automatisms, including lip licking and autonomic signs, such as unilateral hypersalivation characterized by paroxysmal drooling of clear, non-foamy saliva from the right commissure of the mouth, leaving the hair on the ipsilateral lip visibly wet (Supplementary Video 1).

**Figure 1:**
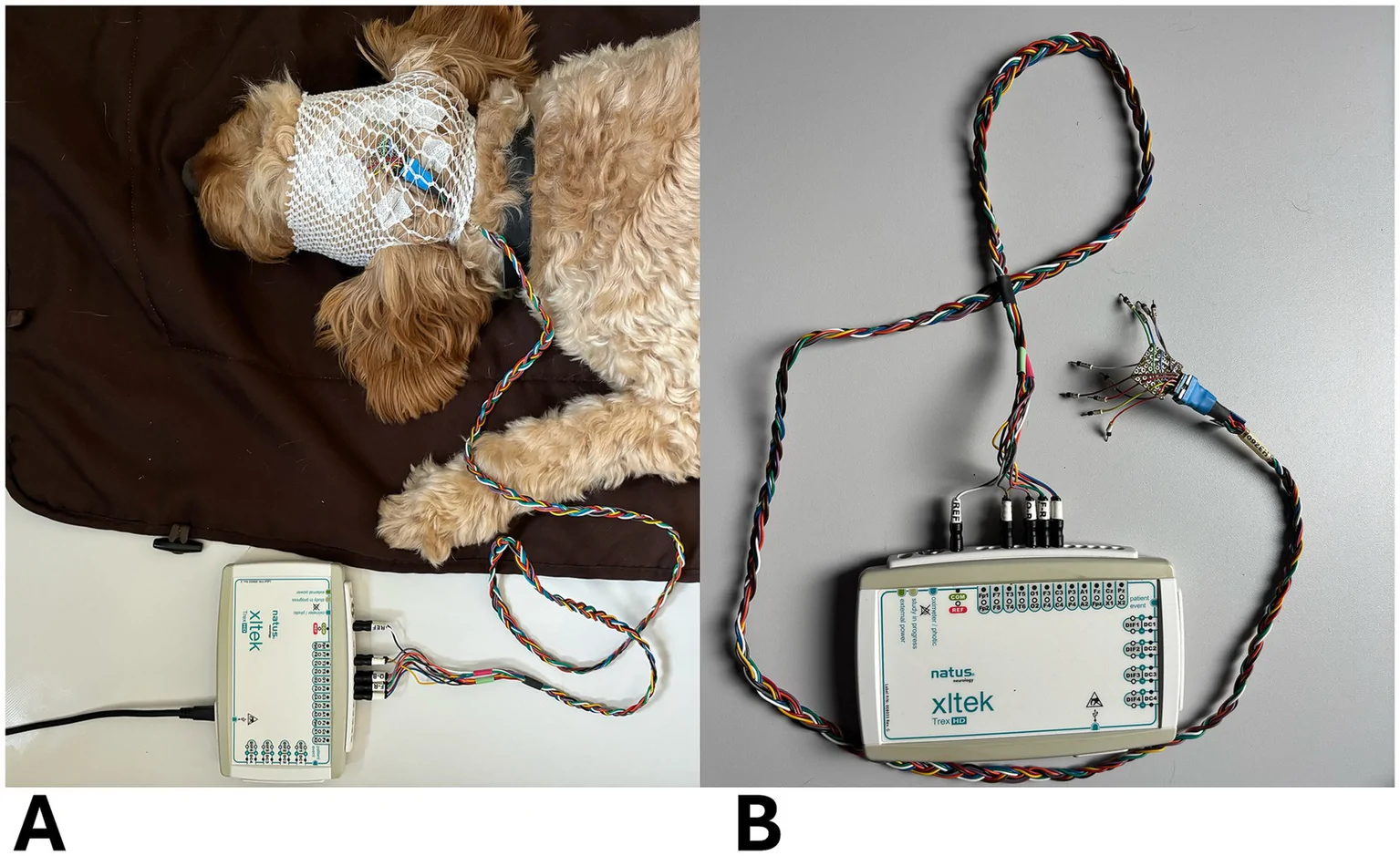
The wireless EEG recording system used in the present study. **(A)** Dog prepared for EEG recording with subdermal wire electrodes secured using a tubular elastic bandage and connected to the wireless EEG recorder. **(B)** Wireless EEG recording unit (Natus Xltek) used for long-term EEG monitoring. Image courtesy of Natus Medical Incorporated - natus.com.

Each episode lasted a few minutes and occurred multiple times per day. In the first and second cases, the episodes occurred almost every day, and in the third case, every 1–2 weeks. No pre-ictal or post-ictal symptoms were observed, and the owners were unable to interrupt or distract the dogs during the episodes. Clinical and neurological examinations were unremarkable in all three cases. The dogs were subjected to a 1.5–4 h wireless electroencephalographic study using a previously described protocol (8) (Figure 1). Medetomidine (20mcg/kg, administered IM into the right triceps muscle) was used solely to facilitate placement of the subdermal wire electrodes. EEG recording was initiated immediately after electrode placement. The dogs gradually recovered from sedation during the recording and typically regained wakefulness within approximately 20–30 min after medetomidine administration. Consequently, the majority of the EEG recording was obtained while the dogs were awake, although some dogs occasionally became drowsy or fell asleep spontaneously during the prolonged recording. Recordings were continued for as long as the dog tolerated the procedure, which accounted for the variation in recording duration. The recordings were performed prior to general anesthesia, necessary for magnetic resonance imaging (MRI) and CSF examinations. The examined dogs were placed in sternal recumbency.

The recordings were carried out with the use of an EEG unit (Natus Xltek) using the following settings: sensitivity 10–15 μV/mm; band pass filter 0.5–0.30 Hz; a 0.3 s time constant; and an inserted 50-Hz notch filter. Each recording was performed using eight active recording electrodes (F3, F4, C3, C4, T3, T4, O1, and O2), one reference electrode placed on the frontal bone, and one ground electrode placed over the occipital region (Figure 1). The recordings were reviewed using a referential montage and a standard bipolar montage (F3-C3, C3-T3, T3-O1, F4-C4, C4-T4, and T4-O2). The EEG recordings were visually inspected for movement, muscle, electrode, and other technical artifacts, which were excluded before interpretation of epileptiform activity. Although synchronized video-EEG recording was not available, all dogs were continuously observed by a veterinary neurologist (AO, AC, DF) throughout the EEG examination, allowing correlation of any overt clinical signs with the EEG recording. All EEG recordings were subjected to visual analysis. They were first reviewed by one veterinary neurologist (AC), and subsequently, a second veterinary neurologist (AO) performed a blinded analysis using both referential and simultaneous bipolar montage recordings. The visual analysis accounted for physiologic superimposed transients (SITs) and pathologic epileptiform discharges (EDs). Currently accepted (9) nomenclature was used to identify EDs, as described elsewhere (8). After EEG, MRI of the brain was performed using a 1.5 Tesla scanner (Siemens). The IVETF protocol (10) was used to scan the dogs’ brains, including 3D T1-weighted, T2-weighted, FLAIR, and post-contrast 3D T1-weighted sequences, along with either a T2* sequence or susceptibility-weighted imaging (SWI) sequence, depending on the MRI protocol used at the time of examination.

Additionally, cerebrospinal fluid (CSF) obtained by cisternal puncture was analyzed for nucleated cell count, total protein concentration, and cytology. Blood tests, including hematology, serum biochemistry, and blood glucose concentration, were also performed. Serum samples were analyzed at Labor Krone (Bad Salzuflen, Germany) using the laboratory’s routine neuronal antibody panel, which included tissue-based indirect immunofluorescence testing followed by cell-based indirect immunofluorescence assays (IgG) for antibodies against NMDAR, GABAA receptor, GABAB receptor, LGI1, CASPR2, mGluR5, AMPAR1/2, and GAD65. Hematology and serum biochemistry were within their respective reference intervals. Hematology and serum biochemistry were within their respective reference intervals. Cerebrospinal fluid analysis revealed a nucleated cell count of <5 cells/μL and a total protein concentration within the reference interval. All diagnostic findings were unremarkable except for the electroencephalographic abnormalities. Fasting or postprandial bile acids and blood ammonia concentrations were not measured because there was no clinical suspicion of a metabolic disorder based on the patients’ history, neurological examination, or routine laboratory findings.

**Figure 2:**
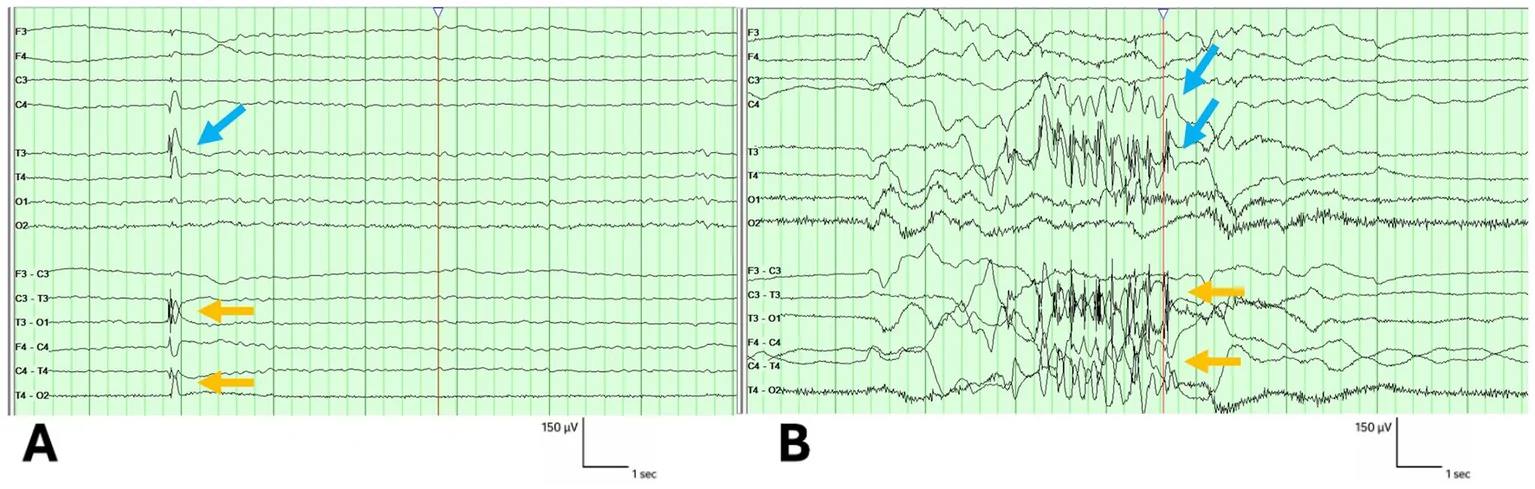
Electroencephalogram recording from a 1.5-h monitoring period in a 2-year-old Border Terrier. The trace demonstrates epileptiform activity, characterized by interictal epileptiform discharges **(A)** Multiple isolated spikes were observed in the monopolar montage over the T3 and T4 leads (blue arrow). Corresponding phase reversals in the bipolar montage were observed between C3-T3, T3-O1 and C4-T4, and T4-O2 (orange arrows). **(B)** Spike–wave complexes were noted in the C4, T3, and T4 leads (blue arrows), with phase reversals in the bipolar montage between C4-T4, T4-O2 and F4-C4, C4-T4 and C3-T3, and T3-O1 (orange arrows). The EEG was recorded using a 10-channel subdermal wire electrode system. Sensitivity: 10 μV/cm; sweep speed: 30 mm/s.

In Case 1, the EEG recording was performed 1 day after the most recent clinical episode. The 90-min EEG recording revealed interictal epileptiform discharges (IEDs) characterized by multiple isolated spikes in the monopolar montage over the T3 and T4 leads, indicating right and left temporal lobe foci. Corresponding phase reversals in the bipolar montage were observed between C3-T3, T3-O1 and C4-T4, and T4-O2. Spike–wave complexes were noted in the C4, T3, and T4 leads, with phase reversals in the bipolar montage between C4-T4, T4-O2 and F4-C4, C4-T4 and C3-T3, and T3-O1 (Figure 2). These findings suggest interictal epileptiform activity originating from the bilateral temporal and right parietal regions, with evidence of synchronous involvement of the temporal lobes. The background activity was within normal limits, with no signs of diffuse slowing or asymmetry.

**Figure 3:**
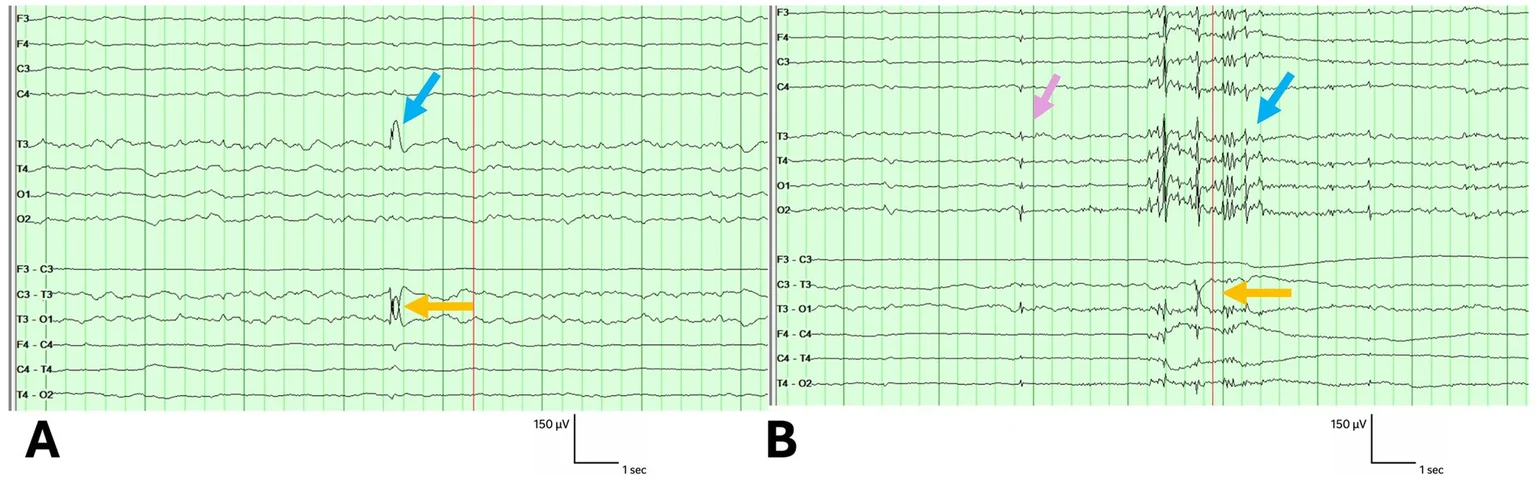
Electroencephalogram recording from a 4-h monitoring period in a 1-year-old Poodle-mix. **(A)** The EEG recording demonstrated an interictal spike-and-wave complex in the monopolar montage over the T3 lead (blue arrow). Corresponding phase reversals in the bipolar montage were observed between C3-T3 and T3-O1 (orange arrow), indicating a left temporal focus. **(B)** In addition, a single generalized EEG discharge was noted, characterized by widespread spike activity across multiple leads in the monopolar montage (blue arrows), with phase reversal localized to T3 (orange arrows). The discharge was preceded by a focal epileptiform spike over the T3 region (pink arrow), suggesting a possible focal onset before secondary generalization. This generalized EEG event was not associated with any observable clinical manifestations. Sensitivity: 15 μV/cm; sweep speed: 30 mm/s.

In Case 2, a 4-h EEG recording performed 3 days after the most recent clinical episode demonstrated IEDs characterized by multiple interictal spike-and-wave complexes in the monopolar montage over the T3 lead. Corresponding phase reversals in the bipolar montage were observed between C3-T3 and T3-O1, indicating a left temporal focus. In addition, a single bilaterally distributed epileptiform discharge was recorded, preceded by a discharge that was maximal over T3, suggesting rapid bilateral propagation or synchronization (Figure 3). These findings are consistent with focal epileptiform activity originating from the left temporal lobe, with evidence of a one-time generalized discharge. The background activity was normal, without any focal or diffuse abnormalities.

**Figure 4:**
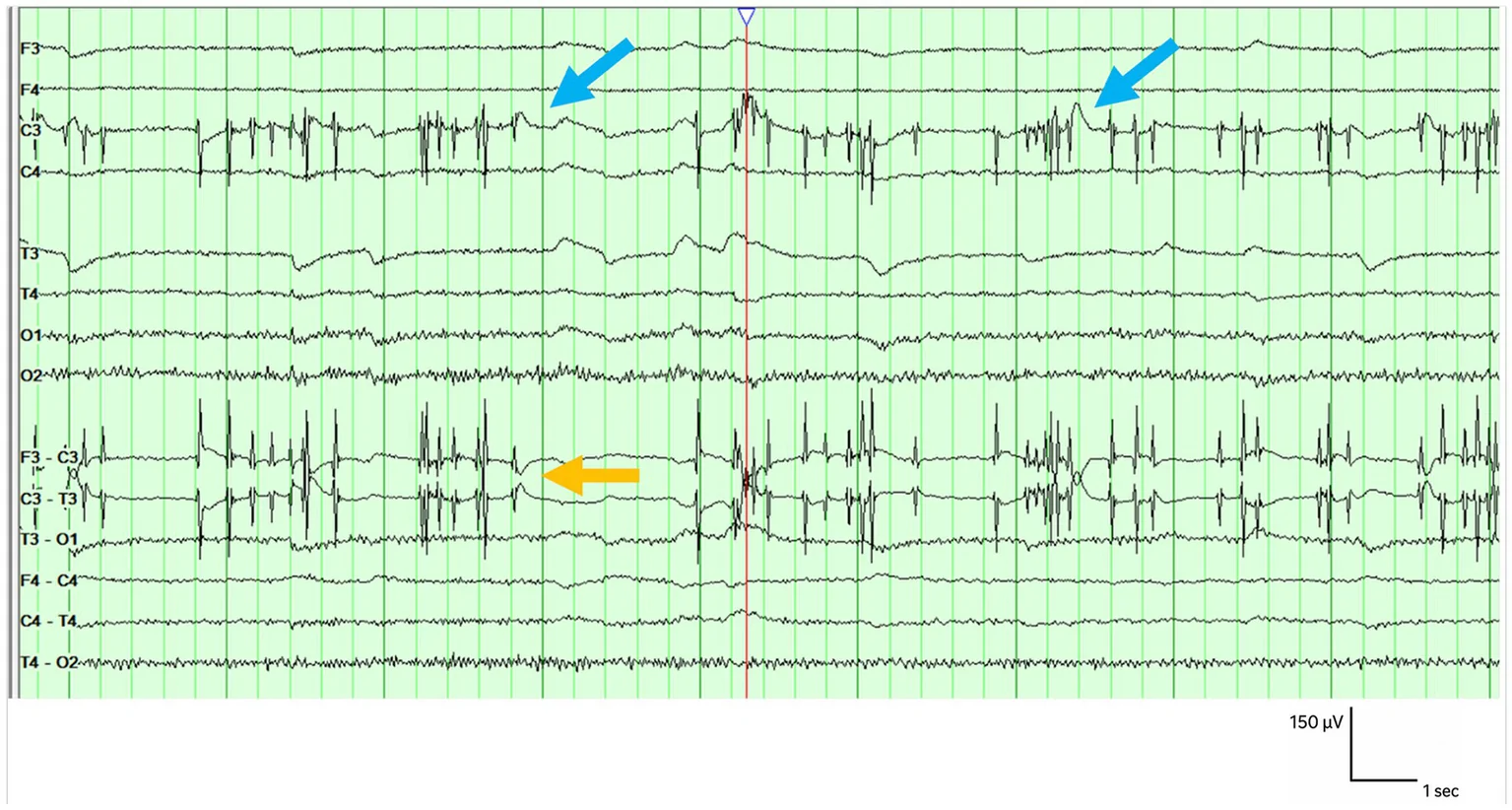
Electroencephalogram recording from a 2.5-h monitoring period in a 4-year-old mixed-breed dog. EEG recording showed multiple, isolated polyspikes and spike waves in the monopolar montage over the C3 lead (blue arrow). Corresponding phase reversals in the bipolar montage were observed between F3-C3 and C3-T3 (orange arrow), indicating a left parietal focus. Sensitivity: 15 μV/cm; sweep speed: 30 mm/s.

In Case 3, a 2.5-h EEG recording performed 1 week after the most recent clinical episode showed multiple, isolated polyspikes and spike waves in the monopolar montage over the C3 lead. Corresponding phase reversals in the bipolar montage were observed between F3-C3 and C3-T3, indicating a left parietal focus (Figure 4). These findings are consistent with focal epileptiform activity originating from the left parietal lobe. The background activity was normal, without any focal or diffuse abnormalities.

No habitual clinical events were observed during EEG recording in any of the three cases. Therefore, the reported EEG findings should be interpreted as interictal epileptiform discharges rather than ictal EEG changes.

All three dogs were treated initially with phenobarbital (approximately 2.5–3 mg/kg PO BID), and they showed a decrease in the frequency of the episodes and a lower intensity. The first case showed a continuous decrease in the frequency of episodes. No more episodes were seen after 4 months of therapy.

After 3 months, the Poodle-mix (second case) again showed a worsening in the frequency of episodes, despite dose adjustments (up to approximately 4 mg/kg PO BID) and phenobarbital serum levels being within the reference range. Levetiracetam (approximately 30 mg/kg PO TID) was added to the phenobarbital therapy, which showed mild improvement, especially in the intensity of the events, although the frequency remained similar. Because the episodes persisted despite the appropriate antiseizure treatment and therapeutic phenobarbital serum concentrations, a concurrent behavioral disorder, including fly-catching syndrome/compulsive behavior, remained among the differential diagnoses. Therefore, fluoxetine (1 mg/kg PO SID) was added, resulting in a brief, modest reduction in event intensity. However, after 1 month, the intensity returned to pre-treatment levels. The owners declined further changes to the treatment with other antiseizure medication (ASM), as they considered the frequency and intensity of the episodes acceptable—reduced from almost daily to approximately once per week—with the combination of phenobarbital and levetiracetam, and the side effects of the therapy were not too severe.

The mixed-breed dog (third case) achieved complete seizure freedom after treatment initiation. A follow-up conducted via phone 3 months later revealed no recurrence of episodes.

## 3 Discussion

Classification of seizure semiology in veterinary medicine is based on the human classification system and includes focal, generalized, and focal-to-generalized seizures (3, 11). In humans, focal seizures are classified as focal aware or focal impaired-awareness seizures and may present with motor or non-motor manifestations, including sensory, cognitive, emotional, behavioral, or autonomic symptoms. Temporal lobe seizures are particularly associated with behavioral and autonomic manifestations, whereas frontal lobe seizures more commonly present with motor signs (4–6, 12, 13).

Temporal lobe epilepsy (TLE) in humans is diagnosed based on a combination of clinical semiology, witness history, EEG findings, and neuroimaging. Typical manifestations include fear, anxiety, confusion, automatisms, altered awareness, olfactory hallucinations, and autonomic signs (13, 14).

In veterinary medicine, non-generalized seizures, particularly focal seizures with impaired awareness, are likely underreported. A recent large-scale study categorized canine seizure semiology into four types: generalized convulsive seizures, non-generalized motor events without convulsions, sudden collapses without movement, and episodes of impaired awareness (7). Episodes characterized by disorientation, unresponsiveness, behavioral arrest, or other subtle behavioral changes may be particularly challenging to classify based on clinical findings alone, highlighting the potential value of EEG as part of the diagnostic workup.

In human medicine, EEG is an important component of the diagnosis of focal epilepsy and contributes to seizure classification and localization when interpreted together with the clinical findings (15). However, scalp EEG has limited spatial resolution, particularly for deep mesial temporal structures (16–20). Longer EEG recordings increase the likelihood of detecting interictal epileptiform activity (19, 20). Similar limitations apply to veterinary medicine, where interpretation of scalp EEG remains challenging due to the lack of a standardized electrode placement system and considerable anatomical variability among breeds.

The present case series describes three dogs that exhibited unusual behavioral manifestations as the sole symptom or along with autonomic signs, such as disorientation, altered responsiveness, and excessive salivation, which are suggestive of focal impaired awareness seizures secondary to idiopathic epilepsy. Interictal epileptiform discharges were recorded over the C3, T3, and T4 electrodes. In conjunction with the clinical semiology, these findings raised suspicion of temporal lobe involvement. While in human medicine these electrodes are reliably associated with mesial temporal activity (16, 17), the situation is more complex in dogs (21–24). Veterinary EEG lacks a universally accepted standardized electrode montage comparable to the human 10–20 system. Moreover, canine skull morphology varies across breeds, and factors such as skull shape, muscle thickness, and brain orientation limit spatial resolution (21, 23, 24). Although the T3/T4 electrodes in dogs are positioned over the zygomatic arch and may correspond to the Sylvian gyrus or temporal lobe, definitive anatomical localization remains presumptive at best (16–18). Thus, while the clinical manifestation and EEG findings in the current cases raise suspicion of temporal lobe epilepsy, this interpretation should be made with caution. These limitations underscore the need to develop breed-specific EEG protocols and advanced imaging or intracranial recording techniques in veterinary neurology. Improved localization would not only aid in the accurate diagnosis of focal epilepsy in dogs but could also refine treatment planning.

In both veterinary and human neurology, routine laboratory tests should also be conducted to rule out toxic or metabolic causes of seizures (4–6, 11). When a cerebral infection or autoimmune process is suspected, a cerebrospinal fluid analysis is warranted to investigate the underlying cause. In humans, an immunological profile for VGKC encephalitis is also regularly performed. This autoimmune condition, causing limbic encephalitis, can also present with cognitive impairment, seizures, and psychiatric symptoms such as hallucinations and aggression (25–27). Similar syndromes have been described in cats, known as complex partial cluster seizures, consisting of orofacial seizures, behavioral changes, and aggression, where VGKC-mediated limbic encephalitis has been reported (28, 29). In dogs, there are only a few reports of positive antibodies against GABA or NMDA (30, 31). In the present cases, serum testing for neuronal antibodies was negative. Although these findings do not exclude an autoimmune etiology, the absence of detectable antibodies, along with normal MRI and CSF findings, makes an inflammatory or autoimmune process less likely and supports a diagnosis of presumed idiopathic epilepsy. Future studies are warranted to evaluate the potential role of routine neuronal antibody screening in dogs presenting with similar clinical signs.

Due to normal blood and CSF results, the therapy consisted only of antiepileptics, in contrast to what is described in cats and humans, also including immunotherapy (32–34). The favorable response to ASM in the first and third cases and the partial response in the second case may provide additional support for an epileptic origin of these events. The described partially satisfactory therapeutic effect is similar to that seen in humans. Many individuals with temporal lobe epilepsy achieve good seizure control with antiseizure drugs. However, treatment response varies depending on the underlying etiology, and approximately one-third of patients develop drug-resistant epilepsy, requiring additional treatment such as temporal lobectomy, selective amygdalohippocampectomy, or, in cases of limbic encephalitis, immunotherapy (35–37). Drug-resistant epilepsy has also been documented in approximately 30% of dogs with idiopathic epilepsy (38). Temporal lobectomy, including amygdalohippocampectomy, represents a promising and developing area in veterinary neurosurgery, with surgical techniques already described for both dogs and cats (39–41). This approach holds potential for treating drug-resistant epilepsy in veterinary patients. Further research is essential to determine the necessity, optimal extent of resection, and efficacy of veterinary temporal lobectomy (VTL), with support from thorough presurgical evaluations and informed consent (42). The antidepressant fluoxetine was added to the treatment in the second case as the patient exhibited symptoms resembling fly-catching behavior, based on reports of better responsiveness to fluoxetine compared to phenobarbital in dogs with fly-catcher syndrome (43). However, in our case, fluoxetine did not produce a satisfactory effect.

A recent case series described four related Labrador retrievers presenting with paroxysmal anxiety or “panic attack-like” episodes associated with focal and generalized epileptic seizures, in which a homozygous missense variant in the *ALDH5A1* gene was identified as a candidate causal variant for a hereditary encephalopathy (44). In contrast to the dogs described in the present study, the affected Labrador retrievers exhibited characteristic bilateral, symmetrical MRI abnormalities, whereas brain MRI findings were unremarkable in all three of our cases. Nevertheless, this article further supports the concept that prominent behavioral manifestations may represent an important component of seizure semiology in dogs with underlying neurological disease. Future studies investigating the genetic basis of dogs presenting with similar behavioral seizure phenotypes, including targeted genetic testing or whole-genome sequencing, may help identify additional hereditary causes and improve our understanding of these disorders.

Taken together, the three dogs shared remarkably similar electroclinical characteristics. All presented predominantly with behavioral manifestations accompanied by autonomic signs, with limited or absent motor signs, normal MRI and CSF findings, and supportive interictal epileptiform discharges on scalp EEG. These observations suggest that focal impaired awareness seizures should be considered in the differential diagnosis of dogs presenting with recurrent paroxysmal behavioral abnormalities, even in the absence of generalized motor seizures.

One limitation of this study is that the residual effects of medetomidine on the early portion of the EEG recording cannot be completely excluded, as α2-adrenoreceptor agonists can influence background EEG activity, although the majority of the recordings were obtained after the dogs had regained wakefulness (45). Additionally, quantitative analysis of interictal epileptiform discharge frequency was not performed, and no habitual clinical events were captured during EEG monitoring. These limitations should be considered when interpreting the electroclinical significance of the EEG findings. Future prospective studies incorporating quantitative EEG analysis together with ictal EEG recordings could provide additional insights into the electroclinical characteristics of canine focal epilepsy.

## 4 Conclusion

This case series describes an uncommon presentation of focal impaired awareness seizures in dogs characterized predominantly by behavioral and autonomic manifestations in the absence of generalized motor seizures. The combination of clinical semiology, diagnostic workup, and supportive interictal EEG findings provided evidence supporting an epileptic origin of these episodes. Diagnostic parallels with human temporal lobe epilepsy highlight the value of EEG and neuroimaging as part of the diagnostic workup in veterinary neurology. These findings emphasize the importance of recognizing alternative seizure manifestations in veterinary patients, improving owner education about the behavioral manifestations of canine epilepsy, and support the inclusion of focal epilepsy among the differential diagnoses of recurrent paroxysmal behavioral abnormalities. Further studies are needed to improve the electroclinical characterization and diagnostic approaches for focal epilepsy in dogs.

## Data Availability

The original contributions presented in the study are included in the article/Supplementary material, further inquiries can be directed to the corresponding author.

## References

[1] Banerjee PN Filippi D Hauser WA. The descriptive epidemiology of epilepsy – a review. Epilepsy Res. (2010) 85:31–45. doi: 10.1016/j.eplepsyres.2009.03.003, 19369037 PMC2696575

[2] Hülsmeyer VI Fischer A Mandigers JJP De Risio L Berendt M Rusbridge C . International veterinary epilepsy task force’s current understanding of idiopathic epilepsy of genetic or suspected genetic origin in purebred dogs. BMC Vet Res. (2015) 11:175. doi: 10.1186/s12917-015-0463-0, 26316206 PMC4552344

[3] Berendt M Farquhar GR Mandigers JJP Pakozdy A Bhatti SFM De Risio L . International veterinary epilepsy task force consensus report on epilepsy definition, classification and terminology in companion animals. BMC Vet Res. (2015) 11:182. doi: 10.1186/s12917-015-0461-2, 26316133 PMC4552272

[4] Ighodaro ET Maini K Arya K Sharma S. "Focal onset seizure". In: StatPearls. Treasure Island, FL: StatPearls Publishing (2025) 29763181

[5] Patel PR De Jesus O. "Partial epilepsy". In: StatPearls. Treasure Island, FL: StatPearls Publishing (2025)

[6] Kumar A Ighodaro ET Sharma S. "Focal impaired awareness seizure". In: StatPearls. Treasure Island, FL: StatPearls Publishing (2025) 30085572

[7] Matz MS Harmas T Wielaender F Hakanen E Nessler JN Volk HA . Development of a novel epilepsy and dyskinesia survey for large-scale characterization of seizure semiology in dogs. J Vet Intern Med. (2025) 39:e70077. doi: 10.1111/jvim.70077, 40375574 PMC12081834

[8] Wrzosek M Ives JR Karczewski M Dziadkowiak E Gruszka E. The relationship between epileptiform discharges and background activity in the visual analysis of electroencephalographic examinations in dogs with seizures of different etiologies. Vet J. (2017) 222:41–51. doi: 10.1016/j.tvjl.2017.03.003, 28410675

[9] Nordli DR Riviello J Niedermeyer E. Seizures and epilepsy in infants to adolescents. In: Schomer DL Silva FL Da, editors. Niedermeyer’s Electroencephalography: Basic Principles, Clinical Applications and Related Fields. Philadelphia: Lippincott (2011). p. 479–540.

[10] Rusbridge C Long S Jovanovik J Milne M Berendt M Bhatti SFM . International veterinary epilepsy task force recommendations for a veterinary epilepsy-specific MRI protocol. BMC Vet Res. (2015) 11:1–15. doi: 10.1186/s12917-015-0466-x, 26319136 PMC4594743

[11] De Risio L Bhatti S Muñana K Penderis J Stein V Tipold A . International veterinary epilepsy task force consensus proposal: diagnostic approach to epilepsy in dogs. BMC Vet Res. (2015) 11:148. doi: 10.1186/s12917-015-0462-1, 26316175 PMC4552251

[12] Fisher RS Cross JH D’Souza C French JA Haut SR Higurashi N . Instruction manual for the ILAE 2017 operational classification of seizure types. Epilepsia. (2017) 58:531–42. doi: 10.1111/epi.13671 28276064

[13] Ladino LD Moien F Tellez-Zenteno JF. "A comprehensive review of temporal lobe epilepsy". In: Neurological Disorders: Clinical Methods, 1st Edn. Hong Kong, China: iConcept Press (2016). p. 1–35.

[14] Cendes F. Mesial temporal lobe epilepsy syndrome: an updated overview. J Epilepsy Clin Neurophysiol. (2005) 11:141–4. doi: 10.1590/S1676-26492005000300006

[15] Lüders HO Najm I Nair D Widdess-Walsh P Bingman W. The epileptogenic zone: general principles. Epileptic Disord. (2006) 8:S1–9. doi: 10.1684/j.1950-6945.2006.tb00204.x 17012067

[16] Niedermeyer E Lopes da Silva FH. Electroencephalography: Basic Principles, Clinical Applications, and Related Fields. Philadelphia: Lippincott Williams & Wilkins (2004).

[17] Saint-Hilaire JM Lee MA. Localizing and lateralizing value of epileptic symptoms in temporal lobe epilepsy. Can J Neuro Sci. (2000) 27:S1–5. doi: 10.1017/s031716710000055x, 10830319

[18] Abou-Khalil B Misulis KE. Atlas of EEG and Seizure Semiology. Philadelphia, PA, USA: Elsevier (2006).

[19] Javidan M. Electroencephalography in mesial temporal lobe epilepsy: a review. Epilepsy Res Treat. (2012) 2012:1–17. doi: 10.1155/2012/637430, 22957235 PMC3420622

[20] Haddad N Melikyan G Alarcon G Shaheen Y Siddiqi M Ali E . 24-hour video EEG in the evaluation of the first unprovoked seizure. Clin Neurophysiol Pract. (2021) 6:123–8. doi: 10.1016/j.cnp.2021.02.006, 33997530 PMC8089767

[21] Keezer MR Simard-Tremblay E Veilleux M. The diagnostic accuracy of prolonged ambulatory versus routine EEG. Clin EEG Neurosci. (2016) 47:157–61. doi: 10.1177/1550059415607108, 26376916

[22] Pellegrino FC Sica RE. Canine electroencephalographic recording technique: findings in normal and epileptic dogs. Clin Neurophysiol. (2014) 115:477–87. doi: 10.1016/S1388-2457(03)00347-X, 14744591

[23] Luca J McCarthy S Parmentier T Hazenfratz M Linden AZ Gaitero L . Survey of electroencephalography usage and techniques for dogs. Front Vet Sci. (2023) 10:1198134. doi: 10.3389/fvets.2023.1198134, 37520003 PMC10374286

[24] James F. "Introduction to electroencephalography". In: De Risio L Platt S, editors. Canine and Feline Epilepsy: Diagnosis and Treatment. Oxfordshire: CABI (2014). p. 325–46.

[25] James FMK Allen DG Bersenas AME Grovum WL Kerr CL Monteith G . Investigation of the use of three electroencephalographic electrodes for long-term electroencephalographic recording in awake and sedated dogs. Am J Vet Res. (2011) 72:384–90. doi: 10.2460/ajvr.72.3.384, 21355742

[26] Parthasarathi UD Harrower T Tempest M Hodges JR Walsh C McKenna PJ . Psychiatric presentation of voltage-gated potassium channel antibody-associated encephalopathy: case report. Br J Psychiatry. (2006) 189:182–3. doi: 10.1192/bjp.bp.105.012864, 16880491 PMC3838945

[27] Hansen N Timäus C. Autoimmune encephalitis with psychiatric features in adults: historical evolution and prospective challenge. J Neural Transm. (2021) 128:1–14. doi: 10.1007/s00702-020-02258-z, 33026492 PMC7815593

[28] Wagner-Altendorf TA Cirkel A Münte TF. Limbic encephalitis due to antineuronal antibodies: neuropsychological aspects. Z Neuropsychol. (2021) 32:24–9. doi: 10.1024/1016-264X/a000315

[29] Flegel T Matiasek K Füsser M Becker LF Böttcher IC Dietzel J . Complex partial seizures with orofacial involvement in 35 cats: MRI changes, cerebrospinal fluid analysis, voltage-gated potassium channel antibodies and survival. J Feline Med Surg. (2025) 27:9. doi: 10.1177/1098612X251365423, 40916498 PMC12417638

[30] Pakozdy A Halasz P Klang A Bauer J Leschnik M Tichy A . Suspected limbic encephalitis and seizure in cats associated with voltage-gated potassium channel (VGKC) complex antibody. J Vet Intern Med. (2013) 27:212–4. doi: 10.1111/jvim.12026, 23278981

[31] Huenerfauth EI Bien CG Bien C Volk HA Meyerhoff N. Case report: anti-GABA a receptor encephalitis in a dog. Front Vet Sci. (2022) 9:886711. doi: 10.3389/fvets.2022.886711, 35812851 PMC9262380

[32] Stafford EG Kortum A Castel A Green L Lau J Early PJ . Presence of cerebrospinal fluid antibodies associated with autoimmune encephalitis of humans in dogs with neurologic disease. J Vet Intern Med. (2019) 33:2175–82. doi: 10.1111/jvim.15616, 31495976 PMC6766506

[33] Yang J Liu X. Immunotherapy for refractory autoimmune encephalitis. Front Immunol. (2021) 12:790962. doi: 10.3389/fimmu.2021.790962, 34975890 PMC8716621

[34] Glantschnigg-Eisl U Klang A Kneissl S Lang B Waters P Irani SR . A feline model of spontaneously occurring autoimmune limbic encephalitis. Vet J. (2023) 296-297:105974. doi: 10.1016/j.tvjl.2023.105974, 36958405

[35] Shan W Mao X Wang X Hogan RE Wang Q. Potential surgical therapies for drug-resistant focal epilepsy. CNS Neurosci Ther. (2021) 27:994–1011. doi: 10.1111/cns.13690, 34101365 PMC8339538

[36] Martlè V Van Ham L Raedt R Vonck K Boon P Bhatti. Non-pharmacological treatment options for refractory epilepsy: an overview of human treatment modalities and their potential utility in dogs. Vet J. (2014) 199:332–9. doi: 10.1016/j.tvjl.2013.09.055 24309438

[37] Wieser HG. Presurgical diagnosis of epilepsies – concepts and diagnostic tools. J Epileptol. (2016) 24:115–40. doi: 10.1515/joepi-2016-0014

[38] Potschka H Fischer A Löscher W Volk HA. Pathophysiology of drug-resistant canine epilepsy. Vet J. (2023) 296-297:105990. doi: 10.1016/j.tvjl.2023.105990, 37150317

[39] Zilli J Kressin M Schänzer A Kampschulte M Schmidt MJ. Partial cortico-hippocampectomy in cats as therapy for refractory temporal epilepsy: a descriptive cadaveric study. PLoS One. (2021) 16:e0244892. doi: 10.1371/journal.pone.0244892, 33449929 PMC7810294

[40] Hasegawa D Saito M Kitagawa M. Neurosurgery in canine epilepsy. Vet J. (2022) 285:105852. doi: 10.1016/j.tvjl.2022.105852 35716888

[41] Hasegawa D Asada R Hamamoto Y Yu Y Kuwabara T Mizoguchi S . Focal cortical resection and hippocampectomy in a cat with drug-resistant structural epilepsy. Front Vet Sci. (2021) 8:719455. doi: 10.3389/fvets.2021.719455, 34355038 PMC8329420

[42] Hasegawa D. Diagnostic techniques to detect the epileptogenic zone: pathophysiological and presurgical analysis of epilepsy in dogs and cats. Vet J. (2016) 215:64–75. doi: 10.1016/j.tvjl.2016.03.005, 27052436

[43] Wrzosek M Płonek M Nicpoń J Cizinauskas S Pakozdy A. Retrospective multicenter evaluation of the “fly-catching syndrome” in 24 dogs: EEG, BAER, MRI, CSF findings and response to antiepileptic and antidepressant treatment. Epilepsy Behav. (2015) 53:184–9. doi: 10.3389/fvets.2024.1402546 26584225

[44] Schofield ES Butler T Goncalves R Pettit L Wyatt S Jenkins CHA . A novel hereditary encephalopathy in four related Labrador retrievers associated with missense variant in the ALDH5A1 gene. J Vet Intern Med. (2026) 40:1. doi: 10.1093/jvimsj/aalaf021 PMC12881967 41742512

[45] Wrzosek M Banasik A Czerwik A Olszewska A Płonek M Stein V. Use of sedation-awakening electroencephalography in dogs with epilepsy. J Vet Intern Med. (2024) 38:2578–89. doi: 10.1111/jvim.17153, 39133769 PMC11423447

